# The morphology of CLL revisited: the clinical significance of prolymphocytes and correlations with prognostic/molecular markers in the LRF CLL4 trial

**DOI:** 10.1111/bjh.14132

**Published:** 2016-05-06

**Authors:** David Oscier, Monica Else, Estella Matutes, Ricardo Morilla, Jonathan C. Strefford, Daniel Catovsky

**Affiliations:** ^1^ Department of Molecular PathologyRoyal Bournemouth HospitalBournemouthUK; ^2^ Division of Molecular PathologyThe Institute of Cancer ResearchLondonUK; ^3^ Cancer SciencesFaculty of MedicineUniversity of SouthamptonSouthamptonUK

**Keywords:** Chronic lymphocytic leukaemia, prolymphocytes, prognostic markers, molecular markers, morphology

## Abstract

Historically, an increase in the percentage and number of circulating prolymphocytes in chronic lymphocytic leukaemia (CLL) has been associated with strong expression of surface immunoglobulin, trisomy 12 and a poor outcome. This study re‐examines the biological and clinical significance of increased peripheral blood prolymphocytes in 508 patients at entry into the randomized UK Leukaemia Research Fund CLL4 trial. It also investigates the associations between increased prolymphocytes and a comprehensive array of biomarkers. 270 patients (53%) had <5% prolymphocytes, 167 (33%) had 5–9%, 60 (12%) had 10–14% and 11 (2%) had ≥15% prolymphocytes. We show that a higher proportion of prolymphocytes (≥10%) was independently associated with *NOTCH1* mutations (*P* = 0·006), absence of 13q deletion (*P* = 0·001), high CD38 expression (*P* = 0·02) and unmutated *IGHV* genes (*P* = 0·01). Deaths due to Richter syndrome were significantly more common amongst patients who had ≥10% vs <10% prolymphocytes (13% vs 2%) respectively (*P* < 0·0001). ≥10% prolymphocytes was also associated with a shorter progression‐free survival (Hazard ratio [HR] 1·50 [95% confidence interval [CI]: 1·16–1·93], *P* = 0·002) and overall survival (HR 1·99 [95% CI: 1·53–2·59], *P* < 0·0001). Our data support the routine examination of blood films in CLL and suggest that a finding of an increased proportion of prolymphocytes may be a trigger for further evaluation of clinical and laboratory features of progressive disease.

Although peripheral blood lymphocytes in chronic lymphocytic leukaemia (CLL) are typically small with clumped chromatin and scanty cytoplasm, it has long been recognized that a subset of patients present with, or acquire, an increased percentage of lymphocytes that are larger with more abundant cytoplasm, nuclear irregularities, lymphoplasmacytoid features and/or one or more prominent nucleoli.

Early studies evaluating the clinical significance of lymphocyte morphology were confounded by difficulties in distinguishing CLL from other chronic lymphoproliferative disorders and gave disparate results (Peterson *et al*, 1975; Dubner *et al*, 1978). However, following the initial observation that increasing refractoriness to treatment may be accompanied by the appearance of prolymphocytes in the blood (Enno *et al*, 1979), a detailed analysis of 300 cases with either CLL (*n* = 258) or B‐cell prolymphocytic leukaemia (PLL) (*n* = 42) identified >55% circulating prolymphocytes as a defining diagnostic criterion for PLL, and CLL cases with 11–55% circulating prolymphocytes (CLL/PL) as having clinical features intermediate between typical CLL and prolymphocytic leukaemia; namely a higher incidence of splenomegaly and higher intensity of surface immunoglobulin expression (SmIg) than in typical CLL (Melo *et al*, 1986). Within the CLL/PL group, those patients with an absolute prolymphocyte count of ≥15 × 10^9^/l were shown to have a shorter overall survival (OS) than those with a lower absolute prolymphocyte count (Melo *et al*, 1987).

Although the adverse prognostic significance of increased prolymphocytes was subsequently confirmed in other studies (Scott *et al*, 1987; Vallespí *et al*, 1991; Criel *et al*, 1997; Oscier *et al*, 1997), the role of morphological examination of blood films in CLL as a guide to prognosis has diminished with the discovery of multiple newer biomarkers.

The Leukaemia Research Fund (LRF) CLL4 trial randomized previously untreated patients to either chlorambucil or fludarabine, alone or in combination with cyclophosphamide (FC) (Catovsky *et al*, 2007). At randomization, differential white blood cell counts were performed and this has provided an opportunity to re‐evaluate both the prognostic significance of nucleolated cells (prolymphocytes and immunoblasts) regarding progression‐free survival (PFS) and OS as well as their correlation with established and recently identified prognostic markers. We found that increased prolymphocytes were associated with markers of poor prognosis and predicted a shorter PFS and OS.

## Patients and methods

In the LRF CLL4 trial 777 patients were randomized between February 1999 and October 2004 to receive chlorambucil, fludarabine or FC. The patients were previously untreated, 25% having Binet stage A‐progressive disease, 45% stage B and 30% stage C. The male:female ratio was 3:1 and the median age was 65 years (range 35–86 years). Clinical follow‐up was to 31 October 2010, and follow‐up for OS for UK patients was to January 2015 (median 11·8 years; range 10·2–15·9 years). In the UK, the deaths of CLL trial patients are flagged and reported to the Clinical Trial Service Unit at Oxford. For 44 surviving patients resident outside the UK, for whom this information was not available, OS was censored at 31 October 2010.

**Figure 1:**
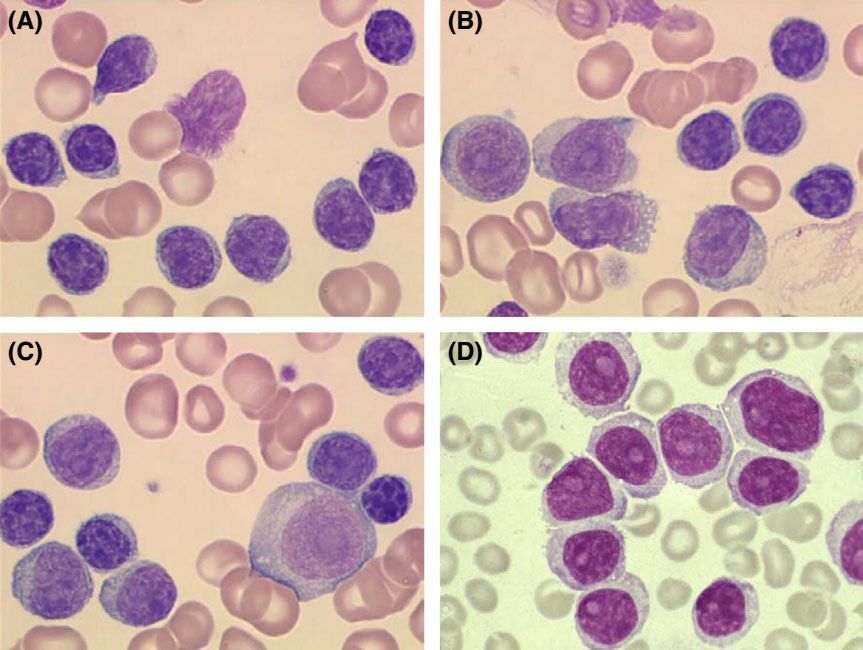
Morphological appearances of chronic lymphocytic leukaemia (CLL) and CLL with >10% circulating prolymphocytes (CLL/PL). (A) Typical CLL. The majority of cells are small with clumped chromatin. (B) Typical CLL/PL. There is a mixture of prolymphocytes and typical CLL lymphocytes. (C) Typical CLL/PL showing small lymphocytes, prolymphocytes and an immunoblast. (D) Typical B‐cell prolymphocytic leukaemia (B‐PLL). The majority of cells have condensed non‐clumped chromatin and a single vesicular nucleolus. This panel is shown here for comparative purposes only, to illustrate the similar morphology of the prolymphocytes in B‐PLL to those seen in panels B and C. The majority of cells in B‐PLL are prolymphocytes and no small lymphocytes are seen. The usual “CLL score” is 0–1. B‐PLL is a distinct disorder and does not arise from a pre‐existing CLL. Magnification: panels A, B and C ×100; panel D ×60.

Differential white blood cell counts were performed on slides stained with May Grunwald Giemsa from blood samples taken at trial entry in 508 patients. A total of 200 cells were scored in patients with lymphocyte counts below 25 × 10^9^/l and 300 cells in the remaining cases. Lymphoid cells were classified according to the criteria of Melo *et al* (1986). Specifically, prolymphocytes were defined as large cells (>2 erythrocytes) with clumped chromatin, a large prominent vesicular nucleolus and usually abundant cytoplasm. Immunoblasts were larger (>3 erythrocytes) with finely dispersed chromatin, large and usually >1 nucleoli, and deeply basophilic cytoplasm (Fig 1). When present, immunoblasts were rare and were included within the prolymphocyte count.

The choice of cut‐off to define an increase in prolymphocytes was based on previous studies of the clinical and biological features of CLL cases with increased prolymphocytes. To determine associations with other variables a 10% cut‐off was used, as this defines the distinction between CLL and CLL/PL. To evaluate the impact on clinical outcome, both a 10% cut‐off and an absolute prolymphocyte count of ≥15 × 10^9^/l were used, as the latter was found to be the best discriminator of outcome within CLL/PL (Melo *et al*, 1987). The clinical value of a 10% cut‐off was also confirmed in our analysis of PFS and OS (see results).

Immunophenotypic analysis was performed centrally by flow cytometry using a panel of monoclonal antibodies: CD5, CD19, CD20, CD23, CD79b, FMC7, and surface light chain immunoglobulins, enabling a CLL score to be derived (Moreau *et al*, 1997). All cases with a low score were also reviewed. Those cases with a phenotype lacking expression of CD23 or CD5, probably reflecting prolonged transit in the postal system or an alternative diagnosis, were only included if lymphocyte morphology was typical of CLL and fluorescent *in situ* hybridization (FISH) for t(11;14) was negative, particularly in CD23 negative cases. Data on the following markers were available: FISH to detect 11q, 13q, 17p deletions and trisomy 12, *IGHV* mutation status, CD38, ZAP70 and *CLLU1* expression, serum beta‐2 microglobulin (B2M), *TP53*,* SF3B1* and *NOTCH1* mutations and telomere length as reported elsewhere, together with a full description of the cut‐offs used to define positivity (Oscier *et al*, 2010, 2013; Gonzalez *et al*, 2011, 2013; Strefford *et al*, 2015).

The LRF CLL4 trial was registered as an International Standard Randomized Trial, number ISRCTN58585610 and was approved by a UK multicentre research ethics committee. The trial followed the UK Medical Research Council guidelines for good clinical practice. All patients provided written informed consent. All authors had access to the primary clinical trial data. The main trial endpoints have been previously reported (Catovsky *et al*, 2007).

Survival was estimated by the Kaplan‐Meier method. OS was calculated from randomization to death from any cause. PFS was estimated from the time of randomization to relapse needing further treatment, progression or death from any cause. For non‐responders (NR) and those with progressive disease (PD), date of progression was when NR/PD was recorded. Multivariate analyses were performed by means of stepwise generalized linear modelling and the Cox proportional hazards model. Values of *P* ≤ 0·05 (two sided) were considered significant. Analyses were performed using the STATISTICA software from StatSoft, a subsidiary of Dell, Inc. (Tulsa, OK, USA).

## Results

Of 508 assessable patients, 270 (53%) had <5% prolymphocytes, 167 (33%) had 5–9%, 60 (12%) had 10–14% and 11 (2%) had ≥15% prolymphocytes. Among the 504 patients in whom an absolute lymphocyte count was available, the absolute prolymphocyte count was <15 × 10^9^/l in 442 (88%) and ≥15 × 10^9^/l in 62 (12%) patients. These groups were equally distributed between the three trial arms. There was a tendency for younger patients, those with stage B disease, a low white blood count, 11q deletion or high *CLLU1* expression to be moderately under‐represented amongst the 508 trial patients who had prolymphocyte data, but otherwise the clinical and molecular characteristics of this subset were the same as those of the 269 patients without available prolymphocyte data (Table SI). Thus the subset with prolymphocyte data was broadly representative of the trial as a whole.

### Association of increased prolymphocytes with immunophenotype

Eighty‐eight per cent of patients had a CLL score of 4 or 5 and 7% had a score of 3. There was no correlation between the CLL score and the percentage of prolymphocytes. However there was a significant association between ≥10% prolymphocytes and strong expression of SmIg (*P* < 0·0001). There was no association between a higher proportion of prolymphocytes and any of the other immunophenotypic markers that comprise the CLL score (Table 1).

**Table 1:** The association between the proportion of prolymphocytes and the CLL score components (atypical phenotypes are shown in bold)

CLL score component	Patients (*n*)	≥10% prolymphocytes (%)	≥15% prolymphocytes (%)	*P*‐value (≥10%)
FMC7 negative	403	59 (15)	7 (2)	NS
**FMC7 positive**	**91**	**10 (11)**	**3 (3)**	
SmIg weak	371	37 (10)	5 (1)	<0·0001^a^
**SmIg strong**	**123**	**32 (26)**	**5 (4)**	
CD23 positive	462	67 (14·5)	10 (2)	NS
**CD23 negative**	**32**	**2 (6)**	**0 (0)**	
CD79b weak	437	59 (13·5)	9 (2)	NS
**CD79b strong**	**57**	**10 (18)**	**1 (2)**	
CD5 positive	490	69 (14)	10 (2)	NS
**CD5 negative**	**4**	**0 (0)**	**0 (0)**	

CLL, chronic lymphocytic leukaemia.

a When CD5‐negative and CD23‐negative cases are excluded, the relationship between surface immunoglobulin (SmIg) expression and ≥10% proplymphocytes remains (10% weak vs 27% strong; *P* < 0·0001).

### Association of increased prolymphocytes with other clinical and laboratory features

Table 2 summarizes the degree of association between ≥10% prolymphocytes and other clinical and laboratory features. No associations were found with treatment arm, age, Binet stage, the presence of lymphadenopathy or splenomegaly, *TP53* deletion or mutation, deletion of 11q, *SF3B1* mutation or ZAP70 expression. Because the variables were available in different sub‐sets of patients, multivariate analysis of the significant variables was performed in consecutive stages, beginning with only the variables that were available from the majority (*n* = 460) of the 508 patients with prolymphocyte data. Absolute prolymphocyte count was not included because of its close relationship, by definition, to % prolymphocytes. Gender, white blood count, 13q deletion and trisomy 12 were each independently significant in this first stage. We then modelled the other significant variables (Table 2) one at a time together with the above four variables. Those which retained independent significance were taken forward to a final model (*n* = 256) which included gender, white blood cell count, *IGHV* mutation status, 13q deletion, trisomy 12, *NOTCH1* mutation and CD38 expression. Four variables were independently associated with percentage of prolymphocytes (Table 3).

**Table 2:** The association of baseline demographic and molecular categorical variables with % prolymphocytes (cut‐off 10%)

Variable		Assessable patients (*n*)	≥10% prolymphocytes (%)	*P*‐value^d^
Randomized first‐line treatment^f^	Chlorambucil	256	34 (13)	NS
	Fludarabine	127	22 (17)	
	FC	125	15 (12)	
Gender^f^	Female	137	11 (8)	0·02
	Male	371	60 (16)	
Age group (years)	<60	154	20 (13)	NS
	60–69	201	26 (13)	
	70+	153	25 (16)	
Disease stage (Binet)	A progressive	139	18 (13)	NS
	B	216	35 (16)	
	C	153	18 (12)	
Splenomegaly	No	221	26 (12)	NS
	Yes	287	45 (16)	
Lymphadenopathy	No	85	12 (14)	NS
	Yes	423	59 (14)	
White blood cell count (cut‐off 100 × 10^9^/l)^f^	Low	256	26 (10)	0·02
	High	246	43 (17)	
*IGHV* mutation status (cut‐off 98%^a^)^f^	Mutated	163	10 (6)	<0·0001
	Unmutated	257	53 (21)	
β‐2 microglobulin (cut‐off 4 mg/l^a^)^f^	Low	201	19 (9)	0·004
	High	169	34 (20)	
*TP53* deletion (cut‐off 10%^a^) or mutation	No	426	60 (14)	NS
	Yes	41	9 (22)	
11q deletion	No	380	59 (16)	NS
	Yes	86	9 (10)	
13q deletion^f^	No	195	43 (22)	0·0001
	Yes	271	25 (9)	
Trisomy 12^f^	No	389	47 (12)	0·0006
	Yes	77	21 (27)	
*NOTCH1* mutation^f^	No	327	39 (12)	<0·0001^e^
	Yes	39	16 (41)	
*SF3B1* mutation	No	284	43 (15)	NS
	Yes	60	13 (22)	
*CLLU1* expression (cut‐off RQ 40^b^)^f^	Low	203	17 (8)	0·0002
	High	194	42 (22)	
CD38 expression (cut‐off 7%^a^)^f^	Negative	163	5 (3)	<0·0001
	Positive	257	54 (21)	
ZAP70 expression (cut‐off 10%^a^)	Negative	191	22 (12)	NS
	Positive	179	31 (17)	
Telomere length^c^, ^f^	Long	79	4 (5)	0·009
	Intermediate	79	13 (16)	
	Short	146	30 (21)	
Absolute prolymphocyte count ≥15 × 10^9^/l	No	442	33 (7)	<0·0001^e^
	Yes	62	36 (58)	

FC, fludarabine with cyclophosphamide.

a Oscier *et al* (2010)

b Gonzalez *et al* (2013); RQ – real time relative quantification

c Cut‐offs defined in Strefford *et al* (2015)

d Chi‐squared test.

e ≥15% vs <15% prolymphocytes also significant: *Notch1 P* = 0·002; absolute prolymphocyte count *P* < 0·0001

f Included in multivariate analysis

**Table 3:** Variables associated with ≥10% prolymphocytes in multivariate analysis

Variable	Odds Ratio	Lower 95% CL	Upper 95% CL	*P*
*NOTCH1* mutation	3.88	1.46	10.30	0.006
Absence of 13q deletion	4.41	1.82	10.69	0.001
Positive CD38 expression	6.48	1.44	29.25	0.02
Unmutated *IGHV* genes	5.02	1.39	18.17	0.01

CL, confidence limit.

### Association of increased prolymphocytes with outcome

There was no significant difference in first‐line overall response rate (74% vs 80%, *P* = 0·3) or first line CR rate (17% vs 18%, *P* = 0·9) for patients with ≥10% vs <10% prolymphocytes respectively.

**Figure 2:**
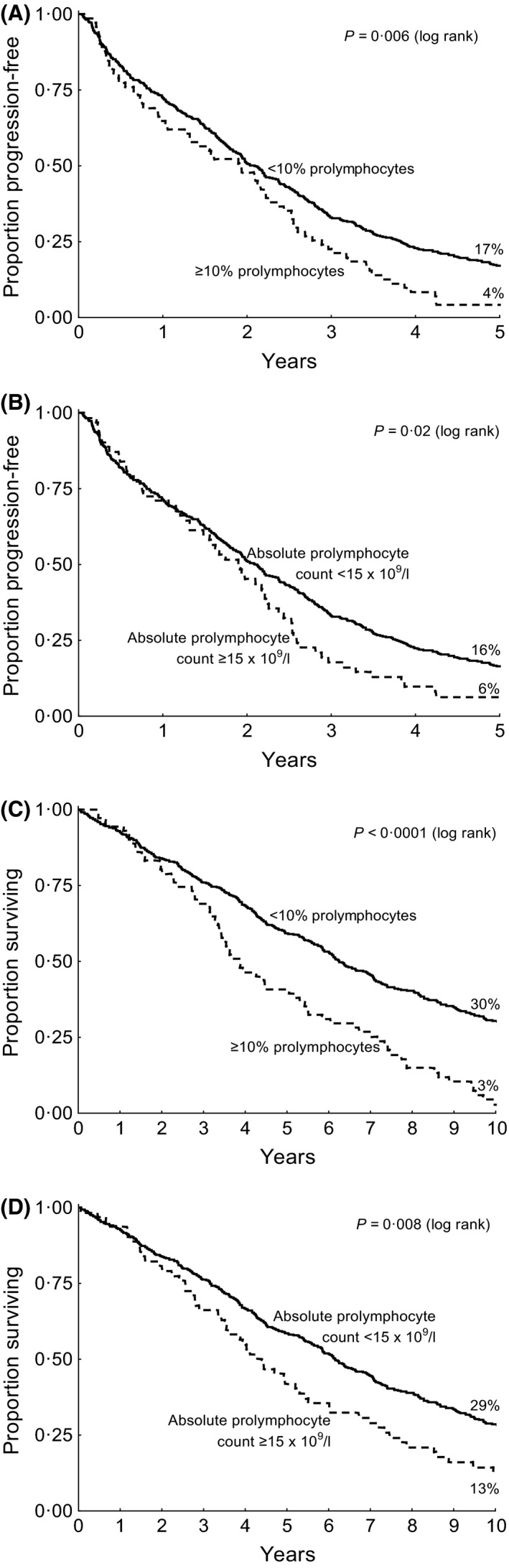
Survival (A) Progression‐free survival by <10% vs ≥10% prolymphocytes. (B) Progression‐free survival by absolute prolymphocyte count. (C) Overall survival by <10% vs ≥10% prolymphocytes (D) Overall survival by absolute prolymphocyte count.

PFS following initial treatment was worse for patients with ≥10% prolymphocytes (Hazard ratio [HR] 1·50 [95% confidence interval (CI): 1·16–1·93], *P* = 0·002) (Fig 2A) and for those with ≥15 × 10^9^/l prolymphocytes (HR 1·45 [95% CI: 1·11–1·91], 0·007) (Fig 2B). The 5‐year PFS was 4% (95% CI: 0–9%) for patients with ≥10% prolymphocytes vs 17% (14–21%) for those with <10% prolymphocytes. OS was also worse for patients with ≥10% prolymphocytes (HR 1·99 [95%CI: 1·53–2·59], *P* < 0·0001) (Fig 2C), and for those with ≥15 × 10^9^/l prolymphocytes (HR 1·53 [95% CI: 1·15 – 2·04], *P* = 0·004) (Fig 2D). OS at 10 years was 3% (0–7%) vs 30% (26–35%) for patients with ≥10% vs <10% prolymphocytes respectively. The adverse prognostic significance of ≥10% prolymphocytes for both PFS and OS was evident in each arm of the trial (Figure S1).

**Figure 3:**
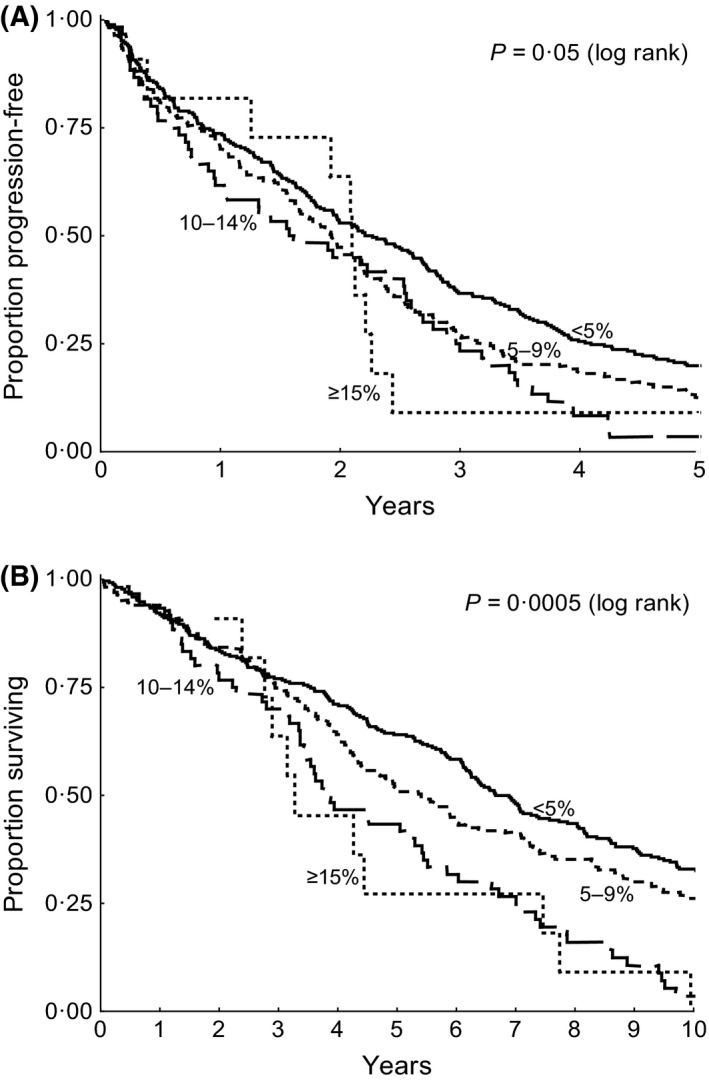
Determining the optimum cut‐off for % prolymphocytes. (A) Progression‐free survival by % prolymphocytes. (B) Overall survival by % prolymphocytes.

Given that the choice of a 10% cut‐off to define increased prolymphocytes was based on historical data, we then compared the PFS and OS for each of four groups defined by % prolymphocytes ranging from <5% to ≥15% (Fig 3). This shows a significant reduction in both PFS and OS compared to cases with <5% prolymphocytes, irrespective of whether the cut‐off was 5% (PFS *P* = 0·0004, OS *P* = 0·0006) or 10% (PFS *P* = 0·006, OS *P* < 0·0001), but provides no support for a cut‐off of >15% (PFS *P* = 0·3, OS *P* = 0·07) which represents only 2% of cases.

Only 8/71 (11%) patients with ≥10% prolymphocytes received no further treatment during the clinical follow‐up period of the trial (median 7 years, range 6–12 years) compared with 146/437 (33%) of those with <10% prolymphocytes (*P* = 0·0002). Two or more further lines of treatment were given to 37/71 (52%) patients with ≥10% prolymphocytes, but to only 134/437 (31%) patients with <10% prolymphocytes (*P* = 0·0004).

To date 17/508 patients (3%) have died as a result of Richter syndrome. Such deaths were significantly more common amongst patients who had ≥10% prolymphocytes at randomization (9/71, 13%) vs those with <10% prolymphocytes (8/437, 2%; *P* < 0·0001).

As with the analysis of factors associated with % prolymphocytes, the multivariate analysis to determine whether % prolymphocytes was independently associated with survival was carried out in three stages, using Cox regression analysis. ≥10% prolymphocytes was an independent predictor of PFS in the first‐stage model (*n* = 465), as were also randomized treatment, gender, 11q deletion and *TP53* deletion/mutation. Adding in the variables from the smaller subsets one at a time, ≥10% prolymphocytes remained an independent predictor of PFS when, in addition to the above first‐stage variables, the model also included any one of the following: ZAP70, *NOTCH1*,* SF3B1* or telomere length, but not when it included any of *IGHV* mutational status, B2M, CD38 or *CLLU1* expression. When all the significant variables from the second stage were included together in a final model (*n* = 120), % prolymphocytes was no longer an independent predictor of PFS.

≥10% prolymphocytes was an independent predictor of OS in the first‐stage model (*n* = 465), as were also disease stage, age, 11q and 13q deletion, and *TP53* deletion/mutation. Adding in the variables from the smaller subsets one at a time, ≥10% prolymphocytes remained an independent predictor of OS when, in addition to the above first‐stage variables, the model also included any one of the following: *IGHV* mutational status, B2M, CD38, ZAP70 or *CLLU1* expression, *NOTCH1* or *SF3B1* mutations or telomere length. When all the significant variables from the second stage were included together in a final model (*n* = 121), % prolymphocytes was no longer an independent predictor of OS.

## Discussion

This is the first study to evaluate the clinical significance of increased circulating prolymphocytes in CLL within the context of a randomized chemotherapy trial and demonstrates that ≥10% prolymphocytes and an absolute prolymphocyte count of ≥15 × 10^9^/l are associated with a shorter PFS and OS in univariate analysis. Independent significance of ≥10% prolymphocytes is lost in multivariate analysis, although it retains significance for OS in models that include either *IGHV* mutational status or B2M, which we previously showed to be independent markers of outcome in the LRF CLL4 trial (Oscier *et al*, 2010). We also noted that patients with ≥10% prolymphocytes were significantly more likely to require second or third line treatments and to die from Richter transformation, although the latter observation requires confirmation in a larger study.

We were able to confirm in this large cohort of patients the previously documented associations between increased prolymphocytes, strong expression of SmIg and trisomy 12, and, for the first time, we document associations with male gender, elevated B2M, unmutated *IGHV* genes, high CD38 and *CLLU1* expression, short telomere length, *NOTCH1* mutations and absence of 13q deletion. Interestingly, and not expected, in a multivariate analysis only unmutated *IGHV* genes, high CD38 expression, *NOTCH1* mutations and absence of 13q deletion, but not trisomy 12, were independently associated with ≥10% prolymphocytes. The association with *NOTCH1* mutations may become stronger once samples are screened for 3′ non‐coding mutations (Puente *et al*, 2015).

Melo *et al* (1986) raised the question as to ‘whether the phenomenon of prolymphocytoid transformation in CLL represents the release into the peripheral blood of cells in the mitotic phase of the cell cycle, or whether the prolymphocytes belong to a subclone with a growth advantage’. With regard to the first possibility, it has subsequently become clear that CLL cells recirculate between secondary lymphoid organs and peripheral blood and the latter contains subpopulations of resting or recently proliferating CLL cells which differ in their expression of surface receptors such as CD38, CD5 and CXCR4.(Calissano *et al*, 2011; Cuthill *et al*, 2015). Within secondary lymphoid organs, CLL cells divide within proliferation centres composed both of tumour cells and components of the tissue microenvironment, such as T cells, monocyte‐derived nurse like cells and stromal cells, with which they interact. (Herishanu *et al*, 2011; ten Hacken & Burger, 2014). Tumour cells within proliferation centres contain medium‐sized and large lymphoid cells comprising prolymphocytes and immunoblasts whose histological features mirror the morphology of circulating prolymphocytes and immunoblasts (Herreros *et al*, 2010). Immunohistochemical studies show that the large tumour cells within proliferation centres show increased expression of Ki67, CD20, CD23, CD38, IRF4, survivin (BIRC5), BCL2 and MYC compared to small lymphocytes outside proliferation centres, and upregulate NOTCH, CD40 and BAFF signalling pathways leading to NF‐kB activation (Patten *et al*, 2008; Giné *et al*, 2010; Herreros *et al*, 2010; Gibson *et al*, 2015; Onaindia *et al*, 2015). Giné *et al* (2010) studied tissue biopsies, mainly from lymph nodes, in 100 CLL patients of whom 73% had suspected Richter transformation. Twenty‐eight per cent had expanded or confluent proliferation centres, which were associated with short survival. Ciccone *et al* (2012) noted a similar association between confluent proliferation centres and short survival in a study of lymph node biopsies in 183 CLL patients. Neither of these studies reviewed lymphocyte morphology in the peripheral blood but, if the emergence of prolymphocytes into the blood represents the egress of cells from proliferation centres with a similar morphological appearance, then both a raised prolymphocyte count and an increase in a subpopulation with the immunophenotype of proliferating cells would be anticipated in these cases.

There are no data to confirm the second possibility that prolymphocytes represent a subclone. However, FISH analysis of proliferation centres and their surrounding areas of small lymphocytes showed a higher incidence of copy number abnormalities within the proliferation centres (Balogh *et al*, 2011). More recently, screening of concurrent blood and lymph node samples for genomic mutations and copy number chromosomal abnormalities identified cases with clonal driver mutations within lymph nodes that were only detected in the blood as small circulating subclones (Del Giudice *et al*, 2016). This supports the concept that mutations arise within a proliferative tissue compartment and raises the possibility that the circulating subclones may be enriched within cells with a ‘proliferative’ immunophenotype and/or those with prolymphocytic morphology.

The association between increased prolymphocytes and *NOTCH1* mutations is a novel and unexpected finding. Circulating CLL cells with trisomy 12 have increased expression of the surface integrins CD11a, CD11b and CD18, which are down‐regulated in the presence of *NOTCH1* mutations (Riches *et al*, 2014). The rare cases of CLL with a *MYC* translocation (Huh *et al*, 2008; Put *et al*, 2012) have been shown to be associated with increased circulating prolymphocytes and although speculative, it is possible that these genomic abnormalities could facilitate the exit of prolymphocytes into the peripheral blood.

Regardless of the biology of prolymphocytes and the reasons for their appearance in the peripheral blood, our study confirms and extends earlier reports of their adverse prognostic significance. Although flow cytometric analysis has become an essential tool in the diagnostic evaluation of CLL and morphological expertise is less widespread, the distinction between cells with or without prominent nucleoli in a well‐made blood film is relatively straightforward. Moreover, we noted that even ≥5% prolymphocytes were associated with a poorer outcome, and the higher cut‐offs used in this study to define increased prolymphocytes clearly distinguish cases with typical morphology from those with CLL/PL. Depending on the clinical context, increased prolymphocytes at diagnosis or during the course of the disease may be a harbinger of progressive disease and may warrant further clinical and laboratory evaluation.

## Author contributions

DC was the principal investigator and takes primary responsibility for the paper; EM and DC evaluated lymphocyte morphology; RM performed flow cytometry, DO and JS undertook the core research on prognostic factors, ME performed the statistical analyses; DC, DO and ME wrote the paper.

## Conflict of interest

The authors have no conflict of interests.

## Supporting information


**Fig S1.** Survival by % prolymphocytes (pl) within each treatment arm A: Progression‐free survival B: Overall survival.
**Table SI.** Prolymphocyte data availability by patient/disease characteristics
