# Supplementary material for: The morphology of CLL revisited: the clinical significance of prolymphocytes and correlations with prognostic/molecular markers in the LRF CLL4 trial
Source: Br J Haematol. 2016 May 6;174(5):767–75. doi: 10.1111/bjh.14132 (PMC4995732; doi:10.1111/bjh.14132)
Supplement: Supplementary file 1 — Fig S1. Survival by % prolymphocytes (pl) within each treatment arm A: Progression‐free survival B: Overall survival. Table SI. Prolymphocyte data availability by patient/disease characteristics [file BJH-174-767-s001.pdf]

Supplementary Table SI: Prolymphocyte data availability by patient/disease characteristics

Variable		Total	Prolymphocyte data available		p-value
			Yes	No	
Randomised first-line treatment	Chlorambucil	387	256 (50)	131 (49)	0.9
	Fludarabine	194	127 (25)	67 (25)	
	FC	196	125 (25)	71 (26)	
Gender	Female	204	137 (27)	67 (25)	0.5
	Male	573	371 (73)	202 (75)	
Age group (years)	<60	255	154 (30)	101 (38)	0.05
	60-69	286	201 (40)	85 (32)	
	70+	236	153 (30)	83 (31)	
Disease stage (Binet)	A progressive	191	139 (27)	52 (19)	0.03
	B	352	216 (43)	136 (51)	
	C	234	153 (30)	81 (30)	
Splenomegaly	No	322	221 (44)	101 (38)	0.1
	Yes	455	287 (56)	168 (62)	
Lymphadenopathy	No	127	85 (17)	42 (16)	0.7
	Yes	650	423 (83)	227 (84)	
White blood cell count (cut-off $100x10^9$/L)	Low	418	256 (51)	162 (61)	0.009
	High	350	246 (49)	104 (39)	
IGHV mutation status (cut-off 98%)	Mutated	206	163 (39)	43 (38)	0.9
	Unmutated	327	257 (61)	70 (62)	
beta-2 microglobulin (cut-off 4mg/L)	Low	309	201 (54)	108 (58)	0.4
	High	247	169 (46)	78 (42)	
TP53 deletion (cut-off 10%) or mutation	No	532	426 (91)	106 (94)	0.4
	Yes	48	41 (9)	7 (6)	
11q deletion	No	463	380 (82)	83 (73)	0.05
	Yes	116	86 (18)	30 (27)	
13q deletion	No	233	195 (42)	38 (34)	0.1
	Yes	346	271 (58)	75 (66)	
Trisomy 12	No	488	389 (83)	99 (88)	0.3
	Yes	91	77 (17)	14 (12)	
Notch1 mutation	No	420	327 (89)	93 (93)	0.3
	Yes	46	39 (11)	7 (7)	
SF3B1 mutation	No	364	284 (83)	80 (86)	0.4
	Yes	73	60 (17)	13 (14)	
CLLU1 expression	Low	247	203 (51)	44 (37)	0.008
	High	268	194 (49)	74 (63)	
CD38 expression	Negative	201	163 (39)	38 (33)	0.3
	Positive	334	257 (61)	77 (67)	
Zap70 expression	Negative	242	191 (52)	51 (47)	0.4
	Positive	236	179 (48)	57 (53)	
Telomere length	Long	96	79 (26)	17 (21)	0.3
	Intermediate	96	79 (26)	17 (21)	
	Short	192	146 (48)	46 (58)	

A

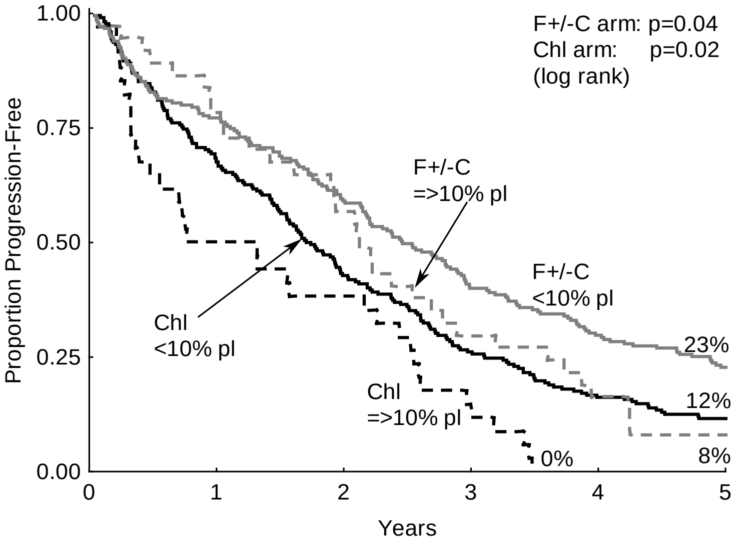

B

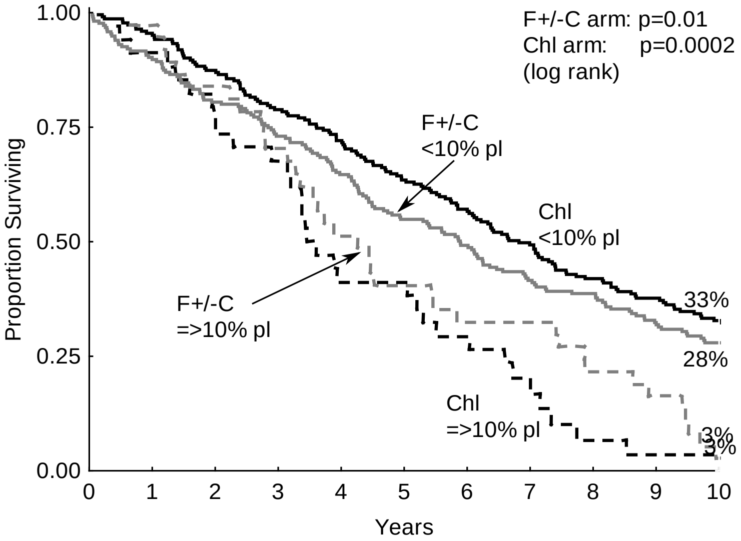

Supplementary Figure S1: Survival by % prolymphocytes (pl) within each treatment arm

A: Progression-free survival

B: Overall survival

Chl – chlorambucil; F+/-C – fludarabine with or without cyclophosphamide
